# 2-(4-Methyl­benzo­yl)benzoic acid monohydrate

**DOI:** 10.1107/S1600536808010581

**Published:** 2008-04-23

**Authors:** Guang-Liang Song, Shui-Ping Deng, Shan Liu, Hong-Jun Zhu

**Affiliations:** a Department of Applied Chemistry, College of Science, Nanjing University of Technology, Nanjing 210009, People’s Republic of China

## Abstract

In the title compound, C_15_H_12_O_3_·H_2_O, the two rings are oriented at a dihedral angle of 69.12 (3)°. In the crystal structure, intermolecular O—H⋯O hydrogen bonds link the mol­ecules into a three-dimensional framework.

## Related literature

For a general background, see: Lin *et al.* (2004 ▶). For a related structure, see: Stanescu (1990 ▶). For bond-length data, see: Allen *et al.* (1987 ▶).
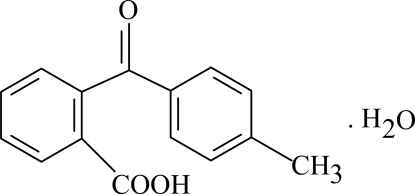


## Experimental

### 

#### Crystal data

C_15_H_12_O_3_·H_2_O
*M* *_r_* = 258.26
Triclinic, 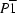

*a* = 7.5410 (15) Å
*b* = 8.7480 (17) Å
*c* = 10.728 (2) Å
α = 79.96 (3)°
β = 77.83 (3)°
γ = 85.63 (3)°
*V* = 680.6 (2) Å^3^
*Z* = 2
Mo *K*α radiation
μ = 0.09 mm^−1^
*T* = 294 (2) K
0.30 × 0.20 × 0.20 mm

#### Data collection

Enraf–Nonius CAD-4 diffractometer
Absorption correction: ψ scan (North *et al.*, 1968 ▶) *T*_min_ = 0.973, *T*_max_ = 0.982
2638 measured reflections
2435 independent reflections
1840 reflections with *I* > 2σ(*I*)
*R*_int_ = 0.052
3 standard reflections frequency: 120 min intensity decay: none

#### Refinement

*R*[*F* ^2^ > 2σ(*F* ^2^)] = 0.061
*wR*(*F* ^2^) = 0.193
*S* = 1.01
2435 reflections
172 parameters
H-atom parameters constrained
Δρ_max_ = 0.39 e Å^−3^
Δρ_min_ = −0.34 e Å^−3^

### 

Data collection: *CAD-4 Software* (Enraf–Nonius, 1985 ▶); cell refinement: *CAD-4 Software*; data reduction: *XCAD4* (Harms & Wocadlo, 1995 ▶); program(s) used to solve structure: *SHELXS97* (Sheldrick, 2008 ▶); program(s) used to refine structure: *SHELXL97* (Sheldrick, 2008 ▶); molecular graphics: *SHELXTL* (Sheldrick, 2008 ▶); software used to prepare material for publication: *SHELXTL*.

## Supplementary Material

Crystal structure: contains datablocks I, global. DOI: 10.1107/S1600536808010581/hk2446sup1.cif

Structure factors: contains datablocks I. DOI: 10.1107/S1600536808010581/hk2446Isup2.hkl

Additional supplementary materials:  crystallographic information; 3D view; checkCIF report

## Figures and Tables

**Table 1:** Hydrogen-bond geometry (Å, °)

*D*—H⋯*A*	*D*—H	H⋯*A*	*D*⋯*A*	*D*—H⋯*A*
O*W*—H*WA*⋯O2^i^	0.85	2.42	2.842 (4)	111
O*W*—H*WB*⋯O1^ii^	0.85	2.38	2.803 (4)	111
O3—H3*B*⋯O*W*	0.82	1.80	2.601 (4)	165

Symmetry codes: (i) 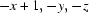; (ii) 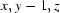.

**Fig. 1.**
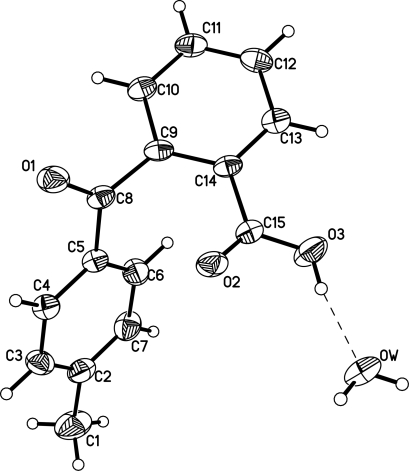
The molecular structure of the title molecule, with the atom-numbering scheme. Displacement ellipsoids are drawn at the 30% probability level. Hydrogen bond is shown as dashed line.

**Fig. 2.**
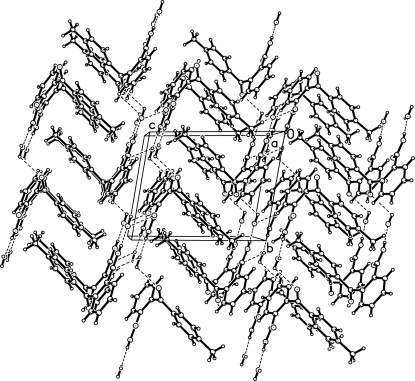
A packing diagram of (I). Hydrogen bonds are shown as dashed lines.

## supplementary crystallographic information

### Comment

2-(4-methylbenzoyl)benzoic acid (MBBA) is an important dye intermediate used for the synthesis of 2-methylanthraquinone (Lin *et al.*,  2004). We report herein the crystal structure of the title compound, (I).

The asymmetric unit of the title compound, (I), (Fig. 1), contains one MBBA and one water molecules. The bond lengths (Allen *et al.*,  1987) and angles are within normal ranges. Rings A (C2-C7) and B (C9-C14) are, of course, planar, and they are oriented at a dihedral angle of 69.12 (3)°.

In the crystal structure, intra- and intermolecular O-H···O hydrogen bonds (Table 1) link the molecules to form a three-dimensional framework (Fig. 2), in which they may be effective in the stabilization of the structure.

### Experimental

The title compound was prepared according to the method described by Stanescu (1990). Crystals of (I) suitable for X-ray analysis were obtained by dissolving MBBA (2.0 g) in water (100 ml) and evaporating water slowly at room temperature for about 15 d.

### Refinement

H atoms were positioned geometrically, with O-H = 0.82 Å (for OH) and 0.85 Å (for H_2_O) and C-H = 0.93 and 0.96 Å for aromatic and methyl H, respectively, and constrained to ride on their parent atoms with U_iso_(H) = xU_eq_(C,O), where x = 1.5 for OH and H_2_O H and x = 1.2 for all other H atoms.

### Crystal data

**Table:**

C_15_H_12_O_3_·H_2_O	*Z* = 2
*M**_r_* = 258.26	*F*_000_ = 272
Triclinic, *P*1	*D*_x_ = 1.260 Mg m^−^^3^
Hall symbol: -P 1	Mo *K*α radiation λ = 0.71073 Å
*a* = 7.5410 (15) Å	Cell parameters from 25 reflections
*b* = 8.7480 (17) Å	θ = 10–13º
*c* = 10.728 (2) Å	µ = 0.09 mm^−^^1^
α = 79.96 (3)º	*T* = 294 (2) K
β = 77.83 (3)º	Block, colorless
γ = 85.63 (3)º	0.30 × 0.20 × 0.20 mm
*V* = 680.6 (2) Å^3^	

### Data collection

**Table:**

Enraf–Nonius CAD-4 diffractometer	*R*_int_ = 0.052
Radiation source: fine-focus sealed tube	θ_max_ = 25.2º
Monochromator: graphite	θ_min_ = 2.0º
*T* = 294(2) K	*h* = −8→9
ω/2θ scans	*k* = −10→10
Absorption correction: ψ scan(North *et al.*, 1968)	*l* = 0→12
*T*_min_ = 0.973, *T*_max_ = 0.982	3 standard reflections
2638 measured reflections	every 120 min
2435 independent reflections	intensity decay: none
1840 reflections with *I* > 2σ(*I*)	

### Refinement

**Table:**

Refinement on *F*^2^	Secondary atom site location: difference Fourier map
Least-squares matrix: full	Hydrogen site location: inferred from neighbouring sites
*R*[*F*^2^ > 2σ(*F*^2^)] = 0.061	H-atom parameters constrained
*wR*(*F*^2^) = 0.193	*w* = 1/[σ^2^(*F*_o_^2^) + (0.07*P*)^2^ + *P*] where *P* = (*F*_o_^2^ + 2*F*_c_^2^)/3
*S* = 1.01	(Δ/σ)_max_ < 0.001
2435 reflections	Δρ_max_ = 0.39 e Å^−^^3^
172 parameters	Δρ_min_ = −0.34 e Å^−^^3^
Primary atom site location: structure-invariant direct methods	Extinction correction: none

### Special details

**Table:**

Geometry. All e.s.d.'s (except the e.s.d. in the dihedral angle between two l.s. planes) are estimated using the full covariance matrix. The cell e.s.d.'s are taken into account individually in the estimation of e.s.d.'s in distances, angles and torsion angles; correlations between e.s.d.'s in cell parameters are only used when they are defined by crystal symmetry. An approximate (isotropic) treatment of cell e.s.d.'s is used for estimating e.s.d.'s involving l.s. planes.
Refinement. Refinement of F^2^ against ALL reflections. The weighted R-factor wR and goodness of fit S are based on F^2^, conventional R-factors R are based on F, with F set to zero for negative F^2^. The threshold expression of F^2^ > 2sigma(F^2^) is used only for calculating R-factors(gt) etc. and is not relevant to the choice of reflections for refinement. R-factors based on F^2^ are statistically about twice as large as those based on F, and R- factors based on ALL data will be even larger.

### Fractional atomic coordinates and isotropic or equivalent isotropic displacement parameters (Å^2^)

**Table:**

	*x*	*y*	*z*	*U*_iso_*/*U*_eq_	
O1	0.3154 (3)	0.5779 (3)	0.2474 (3)	0.0606 (7)	
O2	0.3457 (3)	0.2365 (3)	0.1235 (2)	0.0536 (6)	
O3	0.1304 (3)	0.0831 (3)	0.1112 (3)	0.0741 (9)	
H3B	0.2170	0.0218	0.0961	0.111*	
OW	0.3621 (4)	−0.1469 (3)	0.0679 (3)	0.0649 (7)	
HWA	0.4748	−0.1368	0.0625	0.078*	
HWB	0.3245	−0.2325	0.0583	0.078*	
C1	0.3654 (8)	0.0097 (5)	0.7145 (4)	0.0894 (15)	
H1A	0.2658	−0.0581	0.7457	0.134*	
H1B	0.3792	0.0646	0.7818	0.134*	
H1C	0.4749	−0.0505	0.6892	0.134*	
C2	0.3286 (5)	0.1244 (4)	0.6000 (3)	0.0556 (9)	
C3	0.4516 (5)	0.2367 (4)	0.5386 (3)	0.0552 (9)	
H3A	0.5592	0.2396	0.5672	0.066*	
C4	0.4163 (4)	0.3448 (4)	0.4351 (3)	0.0483 (8)	
H4A	0.5005	0.4194	0.3948	0.058*	
C5	0.2585 (4)	0.3428 (3)	0.3915 (3)	0.0426 (7)	
C6	0.1346 (5)	0.2306 (4)	0.4517 (3)	0.0531 (8)	
H6A	0.0271	0.2276	0.4229	0.064*	
C7	0.1713 (6)	0.1227 (4)	0.5550 (3)	0.0599 (10)	
H7A	0.0876	0.0475	0.5947	0.072*	
C8	0.2224 (4)	0.4632 (3)	0.2816 (3)	0.0431 (7)	
C9	0.0626 (4)	0.4530 (3)	0.2224 (3)	0.0412 (7)	
C10	−0.0716 (4)	0.5713 (4)	0.2360 (3)	0.0491 (8)	
H10A	−0.0566	0.6524	0.2780	0.059*	
C11	−0.2265 (4)	0.5683 (4)	0.1873 (3)	0.0537 (9)	
H11A	−0.3156	0.6474	0.1970	0.064*	
C12	−0.2500 (4)	0.4501 (4)	0.1250 (3)	0.0539 (9)	
H12A	−0.3550	0.4489	0.0928	0.065*	
C13	−0.1169 (4)	0.3311 (4)	0.1096 (3)	0.0471 (8)	
H13A	−0.1338	0.2506	0.0675	0.056*	
C14	0.0391 (4)	0.3326 (3)	0.1566 (3)	0.0410 (7)	
C15	0.1881 (4)	0.2129 (3)	0.1288 (3)	0.0438 (7)	

### Atomic displacement parameters (Å^2^)

**Table:**

	*U*^11^	*U*^22^	*U*^33^	*U*^12^	*U*^13^	*U*^23^
O1	0.0532 (14)	0.0482 (14)	0.0769 (17)	−0.0065 (11)	−0.0189 (12)	0.0089 (12)
O2	0.0396 (13)	0.0515 (14)	0.0682 (15)	0.0127 (10)	−0.0078 (11)	−0.0156 (11)
O3	0.0535 (15)	0.0465 (14)	0.124 (2)	0.0084 (11)	−0.0124 (15)	−0.0292 (15)
OW	0.0646 (16)	0.0387 (13)	0.0809 (18)	0.0083 (11)	0.0040 (13)	−0.0081 (12)
C1	0.128 (4)	0.062 (3)	0.071 (3)	0.016 (3)	−0.027 (3)	0.009 (2)
C2	0.077 (3)	0.0425 (18)	0.0462 (19)	0.0114 (17)	−0.0147 (17)	−0.0077 (15)
C3	0.059 (2)	0.062 (2)	0.0473 (19)	0.0106 (17)	−0.0213 (16)	−0.0103 (16)
C4	0.0482 (19)	0.0444 (18)	0.0505 (18)	0.0008 (14)	−0.0093 (15)	−0.0050 (14)
C5	0.0420 (17)	0.0380 (16)	0.0456 (17)	0.0032 (13)	−0.0067 (13)	−0.0059 (13)
C6	0.058 (2)	0.0505 (19)	0.0506 (19)	−0.0074 (16)	−0.0137 (16)	−0.0015 (15)
C7	0.082 (3)	0.0445 (19)	0.051 (2)	−0.0144 (18)	−0.0119 (18)	0.0037 (15)
C8	0.0378 (16)	0.0369 (16)	0.0499 (18)	0.0034 (13)	−0.0020 (13)	−0.0049 (13)
C9	0.0350 (16)	0.0399 (16)	0.0415 (16)	0.0079 (12)	−0.0010 (12)	0.0007 (13)
C10	0.0444 (18)	0.0440 (18)	0.0552 (19)	0.0105 (14)	−0.0036 (15)	−0.0114 (15)
C11	0.0408 (18)	0.056 (2)	0.058 (2)	0.0182 (15)	−0.0038 (15)	−0.0090 (16)
C12	0.0344 (17)	0.065 (2)	0.057 (2)	0.0076 (15)	−0.0078 (15)	−0.0010 (17)
C13	0.0420 (18)	0.0439 (17)	0.0506 (18)	−0.0004 (14)	−0.0039 (14)	−0.0022 (14)
C14	0.0338 (16)	0.0361 (16)	0.0465 (17)	0.0058 (12)	−0.0004 (13)	−0.0012 (13)
C15	0.0453 (19)	0.0362 (16)	0.0479 (18)	0.0055 (13)	−0.0072 (14)	−0.0068 (13)

### Geometric parameters (Å, °)

**Table:**

O1—C8	1.227 (4)	C5—C8	1.494 (4)
O2—C15	1.209 (4)	C6—C7	1.386 (5)
O3—C15	1.303 (4)	C6—H6A	0.9300
O3—H3B	0.8200	C7—H7A	0.9300
OW—HWA	0.8501	C8—C9	1.491 (4)
OW—HWB	0.8500	C9—C10	1.396 (4)
C1—C2	1.505 (5)	C9—C14	1.407 (4)
C1—H1A	0.9600	C10—C11	1.380 (5)
C1—H1B	0.9600	C10—H10A	0.9300
C1—H1C	0.9600	C11—C12	1.366 (5)
C2—C7	1.374 (5)	C11—H11A	0.9300
C2—C3	1.384 (5)	C12—C13	1.396 (5)
C3—C4	1.386 (5)	C12—H12A	0.9300
C3—H3A	0.9300	C13—C14	1.376 (4)
C4—C5	1.370 (4)	C13—H13A	0.9300
C4—H4A	0.9300	C14—C15	1.495 (4)
C5—C6	1.385 (4)		
			
C15—O3—H3B	109.5	C6—C7—H7A	119.3
HWA—OW—HWB	120.0	O1—C8—C9	119.6 (3)
C2—C1—H1A	109.5	O1—C8—C5	119.7 (3)
C2—C1—H1B	109.5	C9—C8—C5	120.4 (3)
H1A—C1—H1B	109.5	C10—C9—C14	119.1 (3)
C2—C1—H1C	109.5	C10—C9—C8	116.5 (3)
H1A—C1—H1C	109.5	C14—C9—C8	124.4 (3)
H1B—C1—H1C	109.5	C11—C10—C9	120.2 (3)
C7—C2—C3	118.2 (3)	C11—C10—H10A	119.9
C7—C2—C1	121.2 (4)	C9—C10—H10A	119.9
C3—C2—C1	120.6 (4)	C12—C11—C10	120.5 (3)
C2—C3—C4	120.8 (3)	C12—C11—H11A	119.8
C2—C3—H3A	119.6	C10—C11—H11A	119.8
C4—C3—H3A	119.6	C11—C12—C13	120.2 (3)
C5—C4—C3	120.6 (3)	C11—C12—H12A	119.9
C5—C4—H4A	119.7	C13—C12—H12A	119.9
C3—C4—H4A	119.7	C14—C13—C12	120.2 (3)
C4—C5—C6	119.2 (3)	C14—C13—H13A	119.9
C4—C5—C8	119.3 (3)	C12—C13—H13A	119.9
C6—C5—C8	121.5 (3)	C13—C14—C9	119.7 (3)
C5—C6—C7	119.8 (3)	C13—C14—C15	119.7 (3)
C5—C6—H6A	120.1	C9—C14—C15	120.4 (3)
C7—C6—H6A	120.1	O2—C15—O3	124.3 (3)
C2—C7—C6	121.5 (3)	O2—C15—C14	122.5 (3)
C2—C7—H7A	119.3	O3—C15—C14	113.3 (3)
			
C7—C2—C3—C4	0.3 (5)	C5—C8—C9—C14	−64.3 (4)
C1—C2—C3—C4	−178.5 (3)	C14—C9—C10—C11	1.1 (5)
C2—C3—C4—C5	0.1 (5)	C8—C9—C10—C11	−178.1 (3)
C3—C4—C5—C6	−0.4 (5)	C9—C10—C11—C12	−0.2 (5)
C3—C4—C5—C8	178.6 (3)	C10—C11—C12—C13	−0.2 (5)
C4—C5—C6—C7	0.3 (5)	C11—C12—C13—C14	−0.3 (5)
C8—C5—C6—C7	−178.7 (3)	C12—C13—C14—C9	1.2 (4)
C3—C2—C7—C6	−0.5 (5)	C12—C13—C14—C15	−174.3 (3)
C1—C2—C7—C6	178.3 (4)	C10—C9—C14—C13	−1.6 (4)
C5—C6—C7—C2	0.2 (5)	C8—C9—C14—C13	177.6 (3)
C4—C5—C8—O1	−13.4 (4)	C10—C9—C14—C15	173.9 (3)
C6—C5—C8—O1	165.5 (3)	C8—C9—C14—C15	−7.0 (4)
C4—C5—C8—C9	172.9 (3)	C13—C14—C15—O2	152.2 (3)
C6—C5—C8—C9	−8.1 (4)	C9—C14—C15—O2	−23.2 (5)
O1—C8—C9—C10	−58.8 (4)	C13—C14—C15—O3	−27.6 (4)
C5—C8—C9—C10	114.9 (3)	C9—C14—C15—O3	156.9 (3)
O1—C8—C9—C14	122.1 (3)		

### Hydrogen-bond geometry (Å, °)

**Table:**

*D*—H···*A*	*D*—H	H···*A*	*D*···*A*	*D*—H···*A*
OW—HWA···O2^i^	0.85	2.42	2.842 (4)	111
OW—HWB···O1^ii^	0.85	2.38	2.803 (4)	111
O3—H3B···OW	0.82	1.80	2.601 (4)	165

Symmetry codes: (i) −*x*+1, −*y*, −*z*; (ii) *x*, *y*−1, *z*.
